# miR-143-3p Inhibits Aberrant Tau Phosphorylation and Amyloidogenic Processing of APP by Directly Targeting DAPK1 in Alzheimer’s Disease

**DOI:** 10.3390/ijms23147992

**Published:** 2022-07-20

**Authors:** Long Wang, Xindong Shui, Yingxue Mei, Yongfang Xia, Guihua Lan, Li Hu, Mi Zhang, Chen-Ling Gan, Ruomeng Li, Yuan Tian, Quling Wang, Xi Gu, Dongmei Chen, Tao Zhang, Tae Ho Lee

**Affiliations:** Fujian Key Laboratory of Translational Research in Cancer and Neurodegenerative Diseases, Institute for Translational Medicine, School of Basic Medical Sciences, Fujian Medical University, Fuzhou 350122, China; wanglong@fjmu.edu.cn (L.W.); xindongshui18@163.com (X.S.); mln2963@163.com (Y.M.); yfxia0919@163.com (Y.X.); ghlan93@126.com (G.L.); lhu0701@163.com (L.H.); zm15861353835@126.com (M.Z.); ganchenling@fjmu.edu.cn (C.-L.G.); hupimao@live.com (R.L.); tian18615526627@163.com (Y.T.); quling502959@163.com (Q.W.); guxi124@fjmu.edu.cn (X.G.); dmchen88@fjmu.edu.cn (D.C.); taozh@fjmu.edu.cn (T.Z.)

**Keywords:** Alzheimer’s disease, death-associated protein kinase 1 (DAPK1), microRNA, miR-143, tau phosphorylation, amyloid precursor protein, beta-amyloid

## Abstract

The neuropathology of Alzheimer’s disease (AD) is characterized by intracellular aggregation of hyperphosphorylated tau and extracellular accumulation of beta-amyloid (Aβ). Death-associated protein kinase 1 (DAPK1), as a novel therapeutic target, shows promise for the treatment of human AD, but the regulatory mechanisms of DAPK1 expression in AD remain unclear. In this study, we identified miR-143-3p as a promising candidate for targeting DAPK1. miR-143-3p directly bound to the 3′ untranslated region of human DAPK1 mRNA and inhibited its translation. miR-143-3p decreased tau phosphorylation and promoted neurite outgrowth and microtubule assembly. Moreover, miR-143-3p attenuated amyloid precursor protein (APP) phosphorylation and reduced the generation of Aβ40 and Aβ42. Furthermore, restoring DAPK1 expression with miR-143-3p antagonized the effects of miR-143-3p in attenuating tau hyperphosphorylation and Aβ production. In addition, the miR-143-3p levels were downregulated and correlated inversely with the expression of DAPK1 in the hippocampus of AD patients. Our results suggest that miR-143-3p might play critical roles in regulating both aberrant tau phosphorylation and amyloidogenic processing of APP by targeting DAPK1 and thus offer a potential novel therapeutic strategy for AD.

## 1. Introduction

Alzheimer’s disease (AD) is defined by a particular group of cognitive or behavioral changes, such as memory loss and impaired cognition [1]. Unfortunately, despite decades of research, few effective strategies have been established to prevent or slow the rate of disease progression [2]. Currently, AD has become a major burden with substantial costs of care, as well as increasing death rates [1]. The hallmark pathologies of AD are intracellular aggregation of hyperphosphorylated tau and extracellular accumulation of beta-amyloid (Aβ) [3,4]. However, the molecular network of AD remains elusive, and there is a dire need for novel therapeutic approaches.

Death-associated protein kinase 1 (DAPK1) is a calcium/calmodulin-regulated serine/threonine kinase that is known to promote apoptosis under various stimuli [5,6]. DAPK1 also regulates cell growth, inflammation, carcinogenesis, and neurodegeneration [6,7,8,9,10,11]. A genome-wide association study indicated that genetic variations in DAPK1 are significantly associated with the late-onset AD [12,13,14,15]. An increase in DAPK1 levels has been found in the brains of the majority of individuals with AD compared with controls [10,11,16]. Notably, DAPK1 may serve as a key regulator of both tangle and plaque pathologies [14]. DAPK1 enhances tau phosphorylation at multiple AD-related sites, suppresses neurite outgrowth, and inhibits the assembly of tubulin into the microtubules [10,17,18,19,20]. DAPK1 facilitates the amyloidogenic processing of amyloid precursor protein (APP) and promotes Aβ secretion [11]. However, how DAPK1 is modulated in AD remains unclear. Most recently, we found that melatonin can directly bind to DAPK1 and decrease the DAPK1 protein levels via a proteasome-dependent pathway to control the tau phosphorylation [21,22]. We also observed that oligomeric and fibrillar Aβ aggregates can increase the expression of DAPK1 via heat shock protein 90-mediated protein stabilization, which results in a tau hyperphosphorylation [23]. Due to its critical roles in modulating both tau and APP, DAPK1 shows promise for treating human AD, and the regulation of DAPK1 expression needs to be elucidated.

MicroRNAs (miRNAs) are predicted to regulate the activity of approximately half of the total protein-coding genes [24]. Insights into the roles of miRNAs in numerous diseases, including cancer, cardiovascular diseases, and neurological diseases, have contributed to the development of miRNA-based therapeutic strategies [25,26]. Up to 70% of miRNAs are found in the human brain and play important roles in AD-related pathologies [27,28,29]. Recent studies have found that miRNA replacement therapies rescue adult hippocampal neurogenesis and memory deficits in AD models [30]. To date, miRNAs have shown diagnostic and therapeutic value in AD [28,31]. Whether miRNAs regulate tau phosphorylation and APP processing through DAPK1 remains unknown.

MicroRNA-143 (miR-143) is part of a bicistronic cluster composed of miR-143 and miR-145, which is located on chromosome 5 position 33 (5q33) in the human genome and has been implicated in the carcinogenesis [32,33]. Recent findings showing the diagnostic potential of hsa-miR-143-3p for AD have inspired many researchers [34,35,36,37]. However, the hsa-miR-143-3p levels in adult brains and the molecular mechanisms of hsa-miR-143-3p in AD pathogenesis remain unclear.

In this study, we aimed to investigate the potential regulatory mechanisms of DAPK1 expression, which is elevated in human AD brains [10,11,16]. Based on our previous findings, the assumed function of DAPK1 in AD is involved in the phosphorylation of both tau and APP [10,11,21,23], and the interactions have also been predicted by a protein–protein interaction network. We verified that hsa-miR-143-3p directly decreased the DAPK1 protein levels by suppressing translation. Moreover, hsa-miR-143-3p mimics (miR-143-3p) reduced the levels of phosphorylated tau at multiple AD-related sites, and miR-143-3p enhanced neurite outgrowth and microtubule assembly. In addition, miR-143-3p decreased APP phosphorylation and Aβ generation. Restoring DAPK1 antagonized the ability of miR-143-3p to decrease tau phosphorylation, APP phosphorylation, and Aβ secretion by targeting DAPK1. Furthermore, the hsa-miR-143-3p levels were decreased and inversely correlated with the expression of DAPK1 in the hippocampal tissues of individuals with AD. Our results first identify the potential roles of hsa-miR-143-3p in modulating AD pathologies, including both tau dysfunction and Aβ aggregation.

## 2. Results

### 2.1. Hsa-miR-143-3p Directly Targets Human DAPK1 to Suppress Its Protein Expression

**Figure 1:**
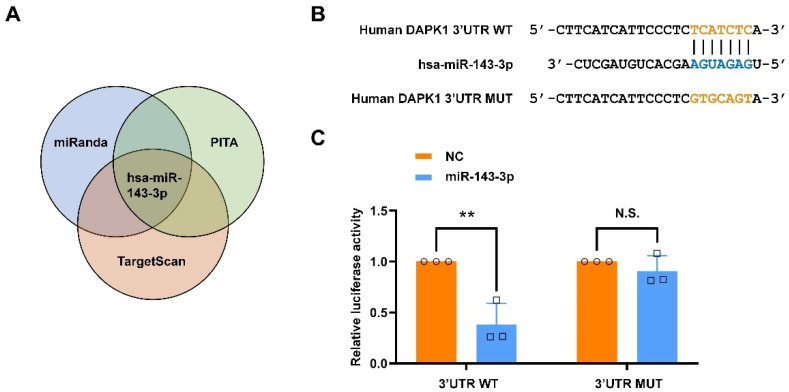
Hsa-miR-143-3p directly targets human DAPK1. (**A**) Hsa-miR-143-3p was predicted as one of the miRNA candidates targeting human DAPK1 using the miRanda, PITA, and TargetScan databases. (**B**) The sequence of the hsa-miR-143-3p WT binding site within the 3′UTR of human DAPK1 mRNA predicted by TargetScan and the sequence of the MUT binding site was cloned into the pmirGLO dual-luciferase vector. (**C**) The relative luciferase activity in HEK 293 cells cotransfected with hsa-miR-143-3p mimics (miR-143-3p) or miRNA negative control (NC) group and the vector including WT or MUT binding sequence was detected by dual-luciferase reporter assays. The data (circles and squares) are presented as the mean ± SD of three independent experiments (** *p* < 0.01, N.S., not significant).

**Figure 2:**
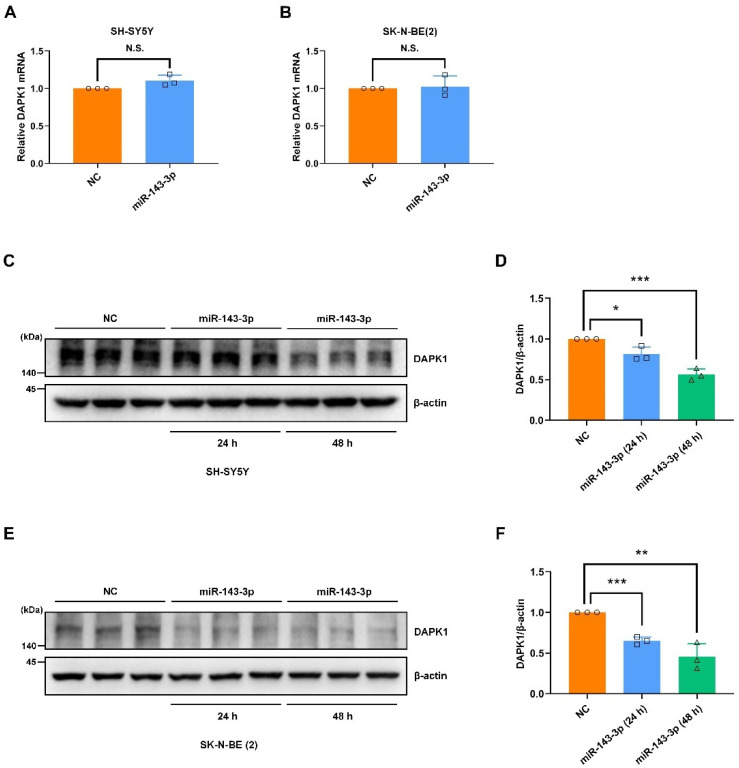
The DAPK1 protein levels are significantly decreased by miR-143-3p. (**A**,**B**) SH-SY5Y (**A**) and SK-N-BE(2) (**B**) cells were treated with miR-143-3p or NC for 24 h, and DAPK1 mRNA expression was analyzed by quantitative RT-PCR. (**C**–**F**) SH-SY5Y (**C**,**D**) and SK-N-BE(2) (**E**,**F**) cells were transfected with NC for 48 h, miR-143-3p for 24 h, or miR-143-3p for 48 h, and DAPK1 protein expression was analyzed by immunoblotting. Each data point (circles, squares and triangles) represents the mean ± SD of three independent experiments (* *p* < 0.05, ** *p* < 0.01, *** *p* < 0.001, N.S., not significant).

Because the DAPK1 levels are aberrantly elevated in patients with AD [10,11,16], we first investigated the molecular mechanisms regulating DAPK1 expression. MiRNAs have been extensively studied as critical post-transcriptional regulatory factors of gene expression and show promise for the diagnosis and treatment of AD [28,30,31]. To determine the potential modulation of DAPK1 expression by miRNAs, we employed bioinformatics databases to predict potential miRNA candidates targeting the 3′ untranslated region (3′UTR) of DAPK1 mRNA. Using three different miRNA online public databases, miRanda, PITA, and TargetScan, we identified hsa-miR-143-3p as a promising candidate potentially targeting human DAPK1 (Figure 1A). To verify whether DAPK1 mRNA directly interacts with hsa-miR-143-3p, we cloned the 3′UTR of human DAPK1 mRNA containing the wild-type (WT) or mutant (MUT) sequence of the predicted binding site into the pmirGLO dual-luciferase vector (Figure 1B). A luciferase activity assay showed that the cotransfection of miR-143-3p with the vector including the WT but not MUT binding sequence significantly suppressed the luciferase activity in the human embryonic kidney (HEK) 293 cells compared with that found for the control group (Figure 1C). Luciferase activity was restored after the sequence of the predicted binding sites was replaced, which suggested that human DAPK1 is a direct target of hsa-miR-143-3p. Furthermore, to determine the regulatory effects of hsa-miR-143-3p on DAPK1, we transfected miR-143-3p or its corresponding control (negative control, NC) into two human neuroblastoma cell lines, SH-SY5Y and SK-N-BE(2). The results showed that the DAPK1 mRNA levels in neuronal cells were not affected by miR-143-3p (Figure 2A,B). However, the DAPK1 protein levels were decreased in 24 h and further reduced in 48 h (Figure 2C–F), demonstrating that miR-143-3p modulated the DAPK1 protein levels through the inhibition of translation instead of mRNA degradation. Taken together, our findings reveal that hsa-miR-143-3p directly targets human DAPK1 and inhibits its protein expression.

### 2.2. Hsa-miR-143-3p Inhibits Tau Phosphorylation

**Figure 3:**
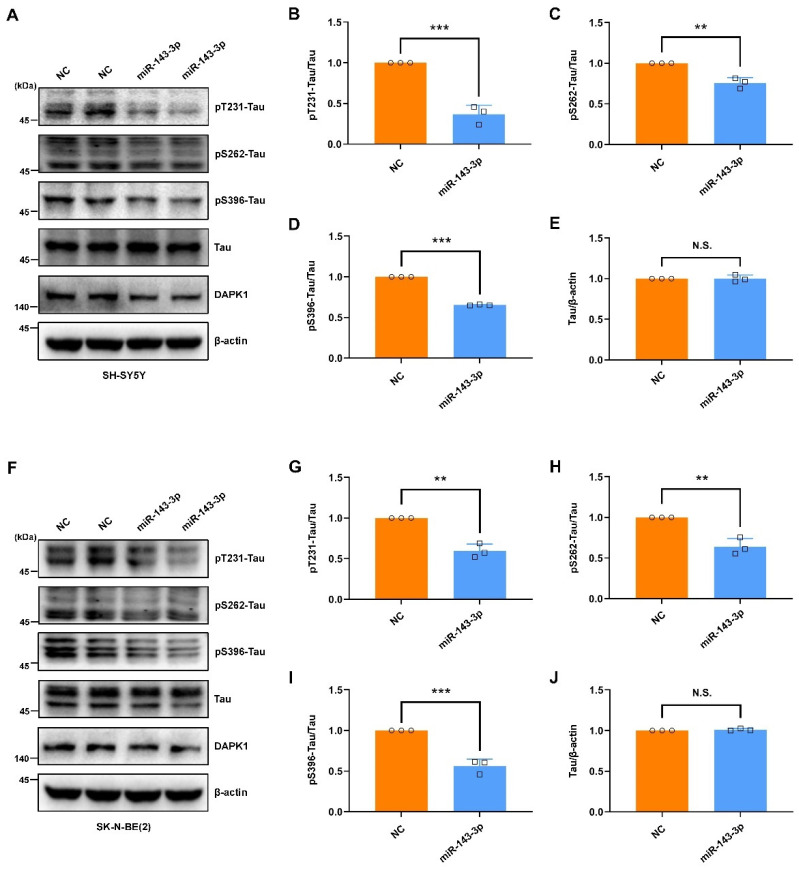
miR-143-3p inhibits tau phosphorylation. (**A**) SH-SY5Y cells were transfected with miR-143-3p or NC for 48 h, and the levels of tau phosphorylated at Thr231, Ser262, and Ser396, total tau, and DAPK1 were analyzed by immunoblotting. (**B**–**E**) Relative quantification was performed using ImageJ software, and the results are presented as a histogram. (**F**) SK-N-BE(2) cells were transfected with miR-143-3p or NC for 48 h, and the levels of tau phosphorylated at Thr231, Ser262, and Ser396, total tau, and DAPK1 were analyzed by immunoblotting. (**G**–**J**) Relative quantification was performed using ImageJ software, and the results are presented as a histogram. The data (circles and squares) are presented as the mean ± SD of three independent experiments (** *p* < 0.01, *** *p* < 0.001, N.S., not significant).

**Figure 4:**
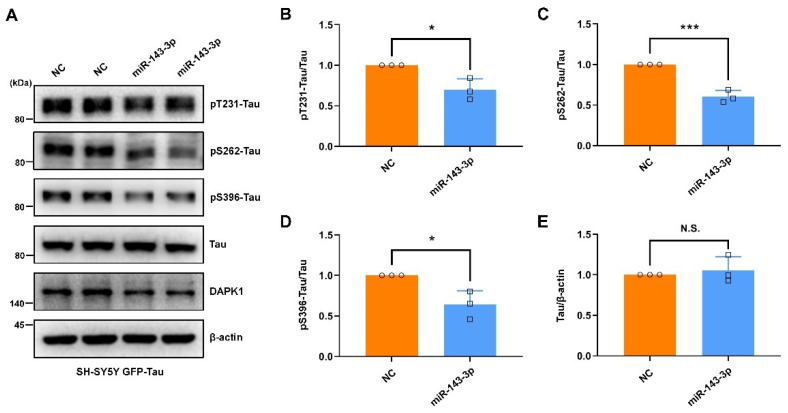
miR-143-3p in SH-SY5Y cells expressing exogenous GFP-tau protein inhibits tau phosphorylation. (**A**) SH-SY5Y cells expressing exogenous GFP-tau protein were transfected with miR-143-3p or NC for 48 h, and the levels of tau phosphorylated at Thr231, Ser262, and Ser396, total tau, and DAPK1 were analyzed by immunoblotting. (**B**–**E**) Relative quantification was determined using ImageJ software, and the results are presented as a histogram. The data (circles and squares) are presented as the mean ± SD of three independent experiments (* *p* < 0.05, *** *p* < 0.001, N.S., not significant).

The functional associations among DAPK1 and AD-associated proteins were constructed by a protein–protein interaction network using the STRING database [38]. The interaction analysis showed that DAPK1 might directly interact with microtubule-associated protein tau (MAPT), APP, and presenilin 1 (PSEN1) (Figure S1), which was consistent with our previous findings [10,11]. Some other critical AD-related proteins, including presenilin 2 (PSEN2), beta-site APP cleaving enzyme-1 (BACE1), a disintegrin and metalloproteinase 10 (ADAM10), and a disintegrin and metalloproteinase 17 (ADAM17), might not be directly modulated by DAPK1. Given the direct modulation of DAPK1 by hsa-miR-143-3p, we further investigated whether hsa-miR-143-3p is implicated in the pathogenesis of AD and its potential therapeutic value. The intracellular aggregation of neurofibrillary tangles (NFTs) consisting of abnormal phosphorylated tau is a major hallmark and is tightly correlated with the severity of cognitive impairment [39,40]. Previous research has indicated that DAPK1 critically affects tau phosphorylation and function, and an increase in DAPK1 results in tau-related pathologies in AD [10,17,18,41]. To investigate the potential roles of hsa-miR-143-3p in tau phosphorylation, we transfected miR-143-3p or NC into cell cultures and measured the levels of both phosphorylated tau at multiple AD-related sites and total tau. The results indicated that miR-143-3p substantially decreased the phosphorylation of tau at Thr231 (pT231-Tau), Ser262 (pS262-Tau), and Ser396 (pS396-Tau) in both SH-SY5Y (Figure 3A–E) and SK-N-BE(2) cells (Figure 3F–J). Moreover, in SH-SY5Y cells expressing exogenous GFP-tau protein, miR-143-3p markedly reduced the levels of phosphorylated tau (Figure 4). However, the total tau levels were not obviously affected in these cells. These observations suggest that hsa-miR-143-3p suppresses the phosphorylation of endogenous and exogenous tau at AD-related sites without influencing the total tau levels.

### 2.3. Hsa-miR-143-3p Promotes Neurite Outgrowth and Microtubule Assembly

**Figure 5:**
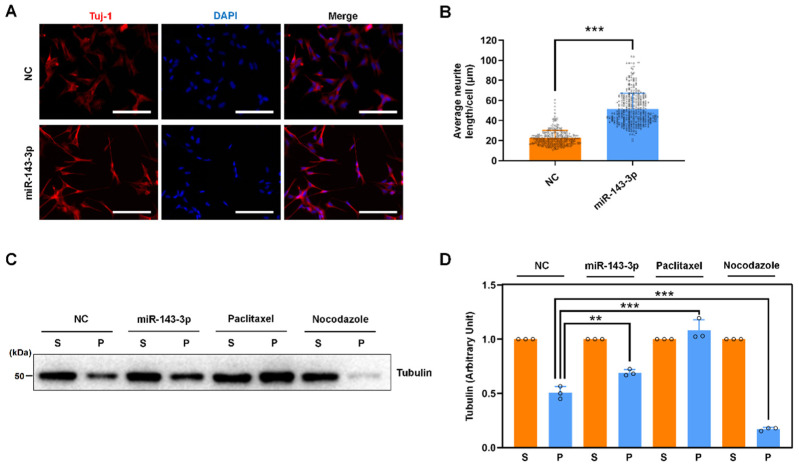
miR-143-3p promotes neurite outgrowth and microtubule assembly. (**A**) SH-SY5Y cells were transfected with miR-143-3p or NC for 24 h prior to cotreatment with RA. The levels of Tuj1 were detected by an immunofluorescence assay. Nuclei were stained with DAPI. Scale bars: 100 μm. (**B**) The average neurite length per cell was quantified in ten random images by ImageJ software and is presented as a histogram. (**C**,**D**) SH-SY5Y cells were transfected with miR-143-3p or NC for 48 h or treated with paclitaxel or nocodazole for 12 h and then lysed with hypotonic buffer. Equal amounts of cytosolic (S, soluble) and cytoskeletal (P, polymerized) proteins were separated by SDS-PAGE, and the levels of tubulin were analyzed by immunoblotting. Relative quantification was performed using ImageJ software, and the results are presented as a histogram. The tubulin expression level in the cytosolic/soluble fraction obtained from each treatment group was arbitrarily defined as ‘1’. The data (circles and squares) are presented as the mean ± SD of three independent experiments (** *p* < 0.01, *** *p* < 0.001).

Tau protein is a family of microtubule-binding proteins that are induced during neurite outgrowth, whereas site-specific phosphorylation modulates microtubule dynamics, neurite outgrowth, and neuronal differentiation, resulting in tau dysfunction in AD [42,43,44,45,46,47,48]. Given that hsa-miR-143-3p inhibits tau phosphorylation, we further explored the biological importance of hsa-miR-143-3p in tau function. miR-143-3p significantly increased retinoic acid (RA)-mediated neuronal differentiation and neurite outgrowth in SH-SY5Y cells, resulting in more cytoplasmic extensions and developed neurites forming distal contacts (Figure 5A,B). Because the best-known function of tau is to promote the assembly of tubulin into microtubules and maintain the structure of microtubules and because the abnormal phosphorylation of tau leads to a disruption of microtubule assembly [42,43,44,45,46,49,50], we also examined the effect of hsa-miR-143-3p on microtubule organization. miR-143-3p increased the protein levels of polymerized tubulin, and microtubule-stabilizing paclitaxel and microtubule-disrupting nocodazole were used as controls (Figure 5C,D). These results indicate that hsa-miR-143-3p may promote neuronal differentiation, neurite outgrowth, and microtubule polymerization by influencing tau functions.

### 2.4. Hsa-miR-143-3p Attenuates APP Phosphorylation and Inhibits the Secretion of Aβ40 and Aβ42

**Figure 6:**
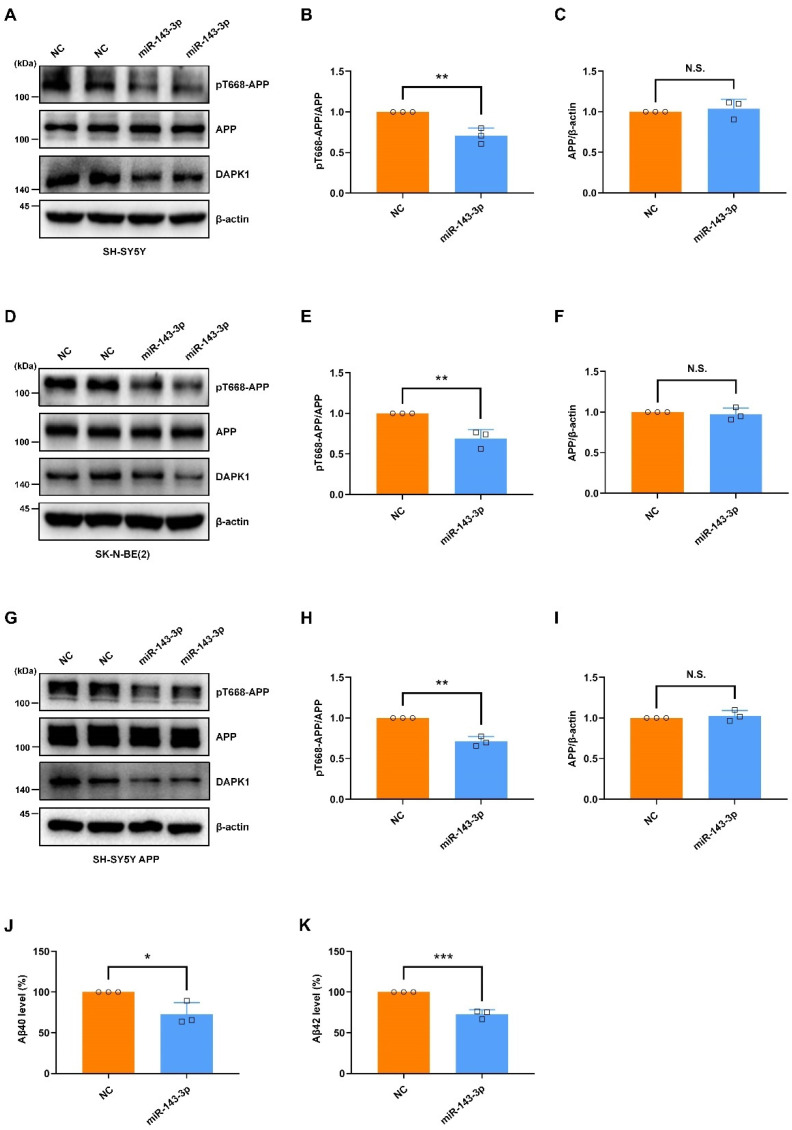
miR-143-3p attenuates APP phosphorylation and inhibits the secretion of Aβ40 and Aβ42. (**A**–**I**) SH-SY5Y (**A**–**C**), SK-N-BE(2) (**D**–**F**), and SH-SY5Y APP (**G**–**I**) cells were transfected with miR-143-3p or NC for 48 h, and the levels of APP phosphorylated at Thr668, total APP, and DAPK1 were analyzed by immunoblotting. (**J**,**K**) SH-SY5Y APP cells were transfected with miR-143-3p or NC for 48 h, and the levels of human Aβ40 (**J**) and Aβ42 (**K**) in cell culture supernatants were determined by a solid-phase sandwich ELISA. Relative quantification was performed using ImageJ software, and the results are presented as a histogram. The data (circles and squares) are presented as the mean ± SD of three independent experiments (* *p* < 0.05, ** *p* < 0.01, *** *p* < 0.001, N.S., not significant).

Another key neuropathological hallmark is extracellular accumulation of Aβ peptide derived from APP [51]. The phosphorylation of APP is critical for APP processing and Aβ generation [23,52]. Notably, APP phosphorylation at Thr668 (pT668-APP) is increased in the hippocampus of AD patients, and this phosphorylation facilitates APP amyloidogenic processing to promote Aβ generation [53]. Our previous study demonstrated that DAPK1 triggers Thr668 phosphorylation of APP and increases Aβ secretion [11]. To determine the roles of hsa-miR-143-3p in APP phosphorylation, we transfected miR-143-3p or NC into cell cultures and measured the levels of APP phosphorylation and total APP. The data revealed that miR-143-3p substantially decreased the levels of APP phosphorylated at Thr668 in SH-SY5Y (Figure 6A–C) and SK-N-BE(2) cells (Figure 6D–F). To confirm these results, we further transfected SH-SY5Y cells stably overexpressing APP (SH-SY5Y APP) with miR-143-3p or NC to evaluate APP phosphorylation. Consistently, miR-143-3p dramatically downregulated the levels of phosphorylated APP in SH-SY5Y APP cells (Figure 6G–I). However, the total APP protein levels were not affected in these cells, indicating that miR-143-3p inhibits the phosphorylation of endogenous and exogenous APP at Thr668 without altering total APP levels. Moreover, to assess the potential effects of miR-143-3p on Aβ generation, we measured the levels of total Aβ40 and Aβ42 secretion in SH-SY5Y APP cells by ELISAs. The data revealed that miR-143-3p strongly suppressed both human Aβ40 and Aβ42 generation (Figure 6J,K). These findings demonstrate that hsa-miR-143-3p attenuates APP phosphorylation at Thr668 and inhibits Aβ40 and Aβ42 secretion.

### 2.5. Restoring DAPK1 Antagonizes the Effects of hsa-miR-143-3p in Attenuating Tau Phosphorylation and Amyloidogenic Processing of APP

**Figure 7:**
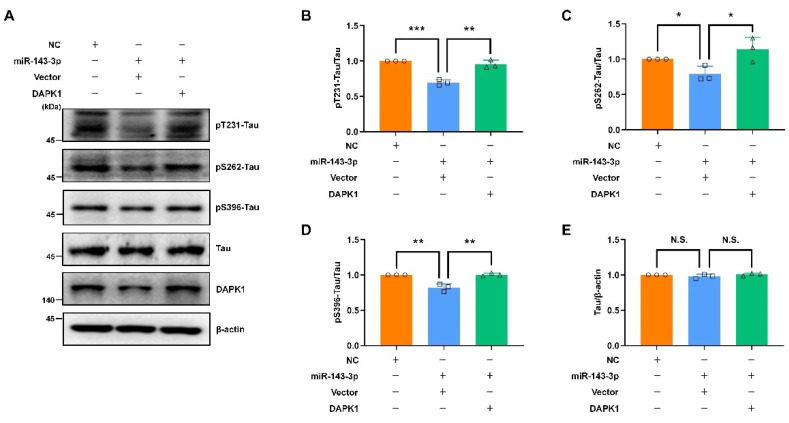
Restoring DAPK1 antagonizes the effects of miR-143-3p in decreasing tau phosphorylation. (**A**) SH-SY5Y cells were transfected with NC, miR-143-3p alone or miR-143-3p together with a plasmid encoding human DAPK1 for 48 h, and the levels of tau phosphorylated at Thr231, Ser262 and Ser396, total tau, and DAPK1 were analyzed by immunoblotting. (**B**–**E**) Relative quantification was performed using ImageJ software, and the results are presented as a histogram. The data (circles, squares and triangles) are presented as the mean ± SD of three independent experiments (* *p* < 0.05, ** *p* < 0.01, *** *p* < 0.001, N.S., not significant).

**Figure 8:**
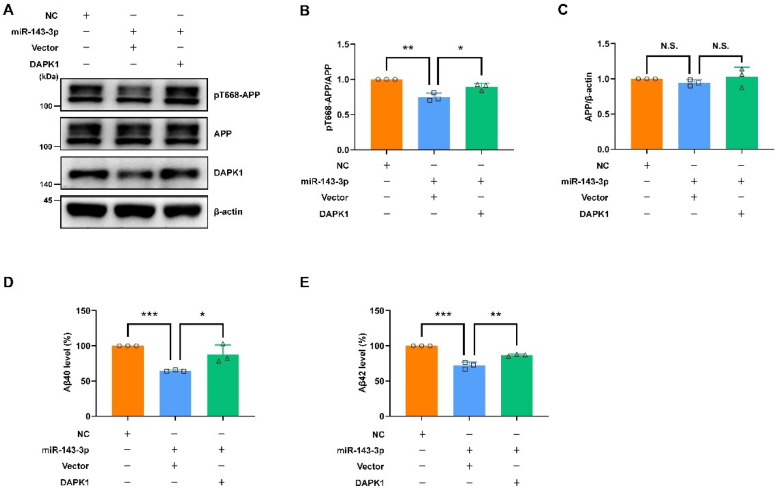
Restoring DAPK1 antagonizes the effects of miR-143-3p in reducing APP phosphorylation and Aβ secretion. (**A**–**C**) SH-SY5Y APP cells were transfected with NC, miR-143-3p alone or miR-143-3p together with a plasmid encoding human DAPK1 for 48 h, and the levels of APP phosphorylated at Thr668, total APP, and DAPK1 were analyzed by immunoblotting. (**D**,**E**) SH-SY5Y APP cells were transfected with NC, miR-143-3p alone or miR-143-3p together with a plasmid encoding human DAPK1 for 48 h, and the levels of human Aβ40 (**D**) and Aβ42 (**D**) in cell culture supernatants were determined by solid-phase sandwich ELISA. Relative quantification was performed using ImageJ software, and the results are presented as a histogram. The data (circles, squares and triangles) are presented as the mean ± SD of three independent experiments (* *p* < 0.05, ** *p* < 0.01, *** *p* < 0.001, N.S., not significant).

To verify the inhibitory roles of hsa-miR-143-3p in tau phosphorylation, APP phosphorylation, and Aβ secretion through the inhibition of DAPK1, we performed rescue experiments via transfection with NC, miR-143-3p alone, or miR-143-3p together with a plasmid encoding human DAPK1 in cell cultures. The results indicated that simultaneous transfection of both miR-143-3p and DAPK1 contributed to a dramatic increase in the levels of phosphorylated tau at Thr231, Ser262, and Ser396 without affecting the total tau levels compared with those found in the groups treated with miR-143-3p alone (Figure 7). Furthermore, APP phosphorylation and Aβ secretion were evaluated. The findings showed that cotransfection of miR-143-3p and DAPK1 significantly induced APP phosphorylation at Thr668 without altering the total APP levels and promoted both human Aβ40 and Aβ42 generation (Figure 8). These data suggest that DAPK1 is sufficient to reverse the inhibitory effects of hsa-miR-143-3p on tau phosphorylation, APP phosphorylation, and both human Aβ40 and Aβ42 secretion.

### 2.6. The hsa-miR-143-3p Levels Are Decreased and Inversely Correlated with the DAPK1 Protein Levels in the Hippocampal Tissues of Patients with AD

**Figure 9:**
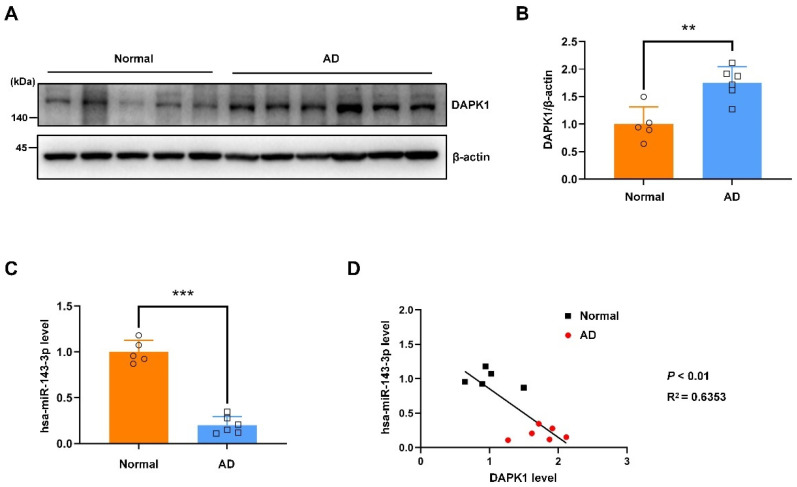
The hsa-miR-143-3p levels are decreased and inversely correlated with DAPK1 expression in the hippocampus of AD patients. (**A**,**B**) Hippocampal tissues of AD patients and age-matched healthy controls were harvested, and the levels of DAPK1 were analyzed by immunoblotting. (**C**) The hsa-miR-143-3p levels in AD and normal control brain tissue samples were analyzed by qRT-PCR using U6 small nuclear RNA as an endogenous control. (**D**) Linear regression analysis was performed to evaluate the correlation between the DAPK1 and hsa-miR-143-3p levels (R^2^ = 0.6353; Pearson’s correlation coefficient). Relative quantification was performed using ImageJ software, and the results are presented as a histogram. The data (circles and squares) are presented as the mean ± SD of three independent experiments (** *p* < 0.01, *** *p* < 0.001).

Our previous findings showed that the expression of DAPK1 is aberrantly elevated in the hippocampus of individuals with AD [10,11,16]. However, the hsa-miR-143-3p levels in adult brains remain elusive. We subsequently aimed to examine the hsa-miR-143-3p levels and clarify whether they are correlated with DAPK1 expression in brain tissue samples. DAPK1 expression was observably elevated in the hippocampus of individuals with AD compared with that of the controls (Figure 9A,B). Of note, the hsa-miR-143-3p levels were dramatically downregulated in the hippocampal samples of individuals with AD compared with age-matched subjects (Figure 9C). Interestingly, marked inverse correlations were found between the DAPK1 and hsa-miR-143-3p levels in human brains, as shown by the Pearson correlation coefficient (R^2^ = 0.6353) (Figure 9D). Collectively, these data reveal that the hsa-miR-143-3p levels are decreased and inversely correlated with DAPK1 expression in the hippocampus of patients with AD.

## 3. Discussion

Although cumulative evidence reveals that DAPK1 plays crucial roles in AD development and that hsa-miR-143-3p is a promising noninvasive biomarker for AD diagnosis, little is known regarding whether and how hsa-miR-143-3p is implicated in AD pathogenesis, and the molecular link between DAPK1 and hsa-miR-143-3p remains unclear. In this study, by mining three different miRNA online databases, we found that hsa-miR-143-3p is one of the overlapping molecules in various intersecting areas, which indicates that it is a promising candidate targeting human DAPK1. The direct negative regulation of DAPK1 by hsa-miR-143-3p through targeting of the 3′UTR of DAPK1 mRNA and inhibition of its translation rather than degradation of the mRNA was further validated. miR-143-3p inhibits the phosphorylation of endogenous and exogenous tau at multiple AD-related sites without influencing the total tau levels. miR-143-3p also promotes neuronal differentiation, neurite outgrowth and microtubule polymerization by regulating tau functions. Furthermore, miR-143-3p attenuates APP phosphorylation and reduces the generation of Aβ40 and Aβ42. Rescue experiments confirmed that miR-143-3p inhibits tau phosphorylation, APP phosphorylation, and Aβ secretion by targeting DAPK1. In addition, in human AD brains, the levels of hsa-miR-143-3p were found to be decreased and inversely correlated with the DAPK1 levels. Taken together, our data illustrate that hsa-miR-143-3p might be a critical regulator of both tau dysfunction and Aβ aggregation by directly targeting DAPK1.

AD pathological changes, including Aβ and tau-mediated neuronal injury and dysfunction, begin to develop decades before the first cognitive symptoms [54,55,56,57]. However, the exact mechanistic link and underlying mechanisms between these two pathological features remain unclear. Our previous findings demonstrated that DAPK1 is aberrantly overexpressed in the hippocampus of individuals with AD and might be a critical regulator of both tau and APP due to its ability to regulate tau function and phosphorylation and control APP processing and Aβ secretion [10,11,14,16]. Importantly, in adult brains, the expression of DAPK1 is restricted to the hippocampus, which is vulnerable to early and severe damage in the cortex [10,58], and this finding suggests that DAPK1 is a potential therapeutic target for AD treatment. Our group recently showed that melatonin interacts with DAPK1 to inhibit protein expression, but a loss of melatonin might not be sufficient to augment the protein stabilization of DAPK1, which indicates that the expression of DAPK1 is modulated by multiple mechanisms, including miRNAs, in AD [14,21]. By employing a variety of miRNA online databases, we identified hsa-miR-143-3p as an attractive and novel biomarker for early AD diagnosis [34,35,36,37]. Through validation using dual-luciferase reporter assays, we provide the first demonstration showing that human DAPK1 is a direct target of hsa-miR-143-3p.

The degree of cognitive dysfunction in AD shows the strongest correlation with the burden of NFTs, whereas abnormal phosphorylation of all six tau isoforms at specific sites gives rise to abnormal filaments [40,59,60,61]. Hyperphosphorylated tau can sequester normal tau, disrupt microtubules, modulate axonal transport, and regulate neurite outgrowth [48,62,63]. Among a variety of phosphorylated tau sites, pThr231 has emerged as the first detectable event during pretangle formation in AD and appears to be key to modulating the conformational changes and misfolding process of the tau [64,65,66]. Moreover, pSer262 within the microtubule-binding region of tau dramatically reduces the binding and might accelerate the formation of tangles from pretangles [67,68,69]. Furthermore, pSer396 has been demonstrated to be one of the earliest events in AD and shows the greatest increase in the frontal cortex [70,71]. Notably, we observed that miR-143-3p significantly suppressed tau phosphorylation at Thr231, Ser262, and Ser396 and promoted neurite outgrowth and microtubule polymerization by inhibiting DAPK1, and these findings provide a potential therapeutic strategy against tau-related pathology in AD. In addition, our findings indicated that the decrease in DAPK1 by miR-143-3p did not affect the total tau levels, which was consistent with previous studies [17]. However, contrasting results have indicated that the levels of endogenous total tau are decreased in DAPK1-knockdown cells [10]. This discrepancy may be due to the difference in DAPK1 reduction induced by short hairpin RNAs (shRNAs) and miRNAs and specific experimental settings, such as the amount and type of RNA used, transfection time, analysis time after transfection, and other transfection conditions. Therefore, further investigations are needed to verify whether miRNA-mediated DAPK1 inhibition affects the tau levels using multiple human cell lines and human primary neurons.

The accumulation of Aβ is hypothesized to be the initiating factor driving AD pathogenesis, whereas monomers of Aβ40 and aggregation-prone and damaging Aβ42 are two predominant forms in the brain [51,72]. Aβ is the product of APP proteolysis via the amyloidogenic pathway [72]. The phosphorylation of APP has been validated as a key regulator of APP processing and Aβ generation [73]. The APP cytoplasmic domain contains eight potential phosphorylation sites that can be targeted by a variety of protein kinases [53,74]. Notably, APP phosphorylation at Thr668 is specific in human brains, is higher in AD patients, and has been demonstrated to increase Aβ secretion by facilitating β-secretase and inducing neurodegeneration by regulating the nuclear translocation of the APP intracellular domain [53,74,75]. Interestingly, our observations revealed that miR-143-3p reduced APP phosphorylation at Thr668 and decreased Aβ generation, thus suggesting a protective function of hsa-miR-143-3p against Aβ pathology in AD.

We identified a direct interaction between human DAPK1 and hsa-miR-143-3p that was predicted by various online databases. According to the miRNA databases, there are conserved sites for miR-143 targeting DAPK1 among vertebrates, including humans, chickens, rhesus macaques, pigs, cats, dogs and other mammals. Surprisingly, the 3′UTR of mouse and rat DAPK1 mRNA did not share the same conserved sites for binding with miR-143. Thus, further validation of our findings was impeded in AD animal models, which mostly consist of transgenic mice. Nonetheless, the vast majority of failures in clinical trials (more than 99%) being in stark contrast to successful preclinical efficacy results using animal models has led to the question of whether animal models truly mirror the pathogenesis of AD [76,77]. Since the most widely used animal models only recapitulate limited pathological features associated with familial AD [76], a better understanding of different molecular mechanisms between humans and mice may help reveal the underlying pathologies of AD and develop potential therapeutics.

Given the particular roles of hsa-miR-143-3p in targeting DAPK1 in human cells, the hsa-miR-143-3p levels in the brain and the potential association between the DAPK1 and hsa-miR-143-3p levels in AD are of interest. In this study, the hsa-miR-143-3p levels were found to be decreased in the hippocampus of individuals with AD compared with the controls. Consistent with these data, other studies showed that hsa-miR-143-3p was dramatically reduced in the serum of individuals with AD relative to the controls and provide a promising noninvasive biomarker for AD diagnosis [35,37]. In contrast, the hsa-miR-143-3p levels have been found to be increased in exosomes isolated from the serum of patients with AD compared with control subjects [34]. Therefore, studies with large sample sizes are needed to clarify the controversial results. We also measured the expression of DAPK1 in the hippocampus, which might suffer from damage earlier and more severely than other regions of the cortex of AD patients [10,58]. The DAPK1 levels were substantially higher in AD brains than in normal brains, which was consistent with previous studies [10,11,16,21]. Interestingly, inverse correlations between DAPK1 protein expression and hsa-miR-143-3p levels were identified, which further demonstrated that hsa-miR-143-3p exerts inhibitory effects on DAPK1 expression.

## 4. Materials and Methods

### 4.1. Cell Culture

The human neuroblastoma cell lines SH-SY5Y and SK-N-BE(2) and HEK 293 cells were obtained from the Stem Cell Bank/Stem Cell Core Facility (Shanghai, China). HEK 293 cells were cultured in high-glucose Dulbecco’s modified Eagle’s medium (DMEM) containing 10% fetal bovine serum (FBS). SH-SY5Y and SK-N-BE(2) cells were cultured in DMEM/F12 with 10% FBS. The cell cultures were maintained at 37 °C under 5% CO_2_.

### 4.2. Plasmids

The 3′UTR sequences of human DAPK1 harboring putative hsa-miR-143-3p-binding sites were first cloned and then inserted into the commercial pmirGLO Dual-Luciferase miRNA Target Expression Vector (E1330, Promega, Madison, WI, USA) using SacI and SalI to generate a DAPK1 wild-type reporter (DAPK1 3′UTR WT). A site-specific mutant reporter in which the putative hsa-miR-143-3p binding sites were mutated (DAPK1 3′UTR MUT) was generated. The coding sequences of human DAPK1 were cloned into the 5′-end-Flag tagged and CMV promoter-driven mammalian expression vector pRK5, as previously described [10].

### 4.3. Transfection

The plasmids, miR-143-3p (B01001, GenePharma, Shanghai, China) and the corresponding controls (GenePharma) were transfected transiently into cells using TurboFect transfection reagent (R0531, Thermo Fisher Scientific, Rockford, IL, USA).

### 4.4. RNA Extraction and Quantitative Real-Time Polymerase Chain Reaction (qRT-PCR) Assay

Total RNA from cells was isolated using NucleoZOL (740404.200, Macherey-Nagel, Dueren, Germany). Single-strand cDNAs were synthesized using a Transcriptor First Strand cDNA Synthesis Kit (04897030001, Roche, Indianapolis, IN, USA). For determination of miRNA levels, a miRNA Extraction Kit (B1802, HaiGene, Harbin, Heilongjiang, China) and One Step miRNA cDNA Synthesis Kit (D1801, HaiGene) were used for miRNA extraction and cDNA synthesis, respectively. qRT-PCR was conducted using a QuantStudio Real-Time PCR system (Applied Biosystems, Waltham, MA, USA) and FastStart Universal SYBR Green Master Mix (04913914001, Roche) as previously described [21]. For the amplification of human DAPK1, DAPK1-F (5′-AGGAACTTGGCAGTGGACAG-3′) and DAPK1-R (5′-CCTTCAGGATGCTGACCTCC-3′) were utilized as primers. For the amplification of human β-actin, β-actin-F (5′-AGGATTCCTATGTGGGCGAC-3′) and β-actin-R (5′-ATAGCACAGCCTGGATAGCAA-3′) were utilized as primers. For hsa-miR-143-3p amplification, hsa-miR-143-3p-F (5′-CAGTGAGATGAAGCACTGTAG-3′) and hsa-miR-143-3p-R (5′-GGTCCAGTTTTTTTTTTTTTTTGAG-3′) were used as primers. For human U6 small nuclear RNA amplification, U6-F (5′-CTCGCTTCGGCAGCACA-3′) and U6-R (5′-AACGCTTCACGAATTTGCGT-3′) were used as primers. The DAPK1 and hsa-miR-143-3p levels were determined using the 2^−ΔΔCt^ method and normalized to the β-actin and U6 levels, respectively.

### 4.5. Immunofluorescence and Immunoblotting Analyses

Immunofluorescence and immunoblotting analyses were performed as previously described [21,23,78,79]. The antibodies used in the study are listed in Table S1. DAPI (E607303, Sangon Biotech, Shanghai, China) was used to stain the nuclei. For immunoblotting analyses, the DAPK1, Tau, and APP levels were measured relative to the levels of the housekeeping protein β-actin. The pT231-Tau, pS262-Tau, and pS396-Tau levels were measured relative to the total tau level. The pT668-APP level was measured relative to the total APP levels.

### 4.6. Luciferase Reporter Assay

HEK 293 cells were cotransfected with DAPK1 3′UTR WT or DAPK1 3′UTR MUT plasmids and miR-143-3p or NC for 48 h and assessed using the Dual-Luciferase Reporter Assay System (E1910, Promega, Madison, WI, USA).

### 4.7. Neurite Outgrowth Assay

For the induction of differentiation, SH-SY5Y cells were cultured in serum-starved conditions (1% FBS) and treated with 10 μM RA (R2625, Sigma, St. Louis, MO, USA) as previously described [80]. The neurite outgrowth levels were measured by enumerating the quantity of neurites and the average neurite length in each cell. Three independent experiments were conducted, and random images were analyzed. Neurites were qualified by comparing their length with the diameter of the cell body [10].

### 4.8. Microtubule Assembly Assay

SH-SY5Y cells were transfected with miR-143-3p or NC for 36 h or exposed to paclitaxel or nocodazole for 12 h [10]. The cells were then lysed with hypotonic buffer (1 mM MgCl_2_, 2 mM EGTA, 20 mM Tris-HCl (pH 6.8), and 0.5% NP-40) containing protease inhibitor cocktail at 37 °C for 5 min and then centrifuged at 18,000× *g* and 25 °C for 10 min. The soluble unpolymerized tubulin in the supernatants was transferred to a new tube. The insoluble polymerized tubulin within the pellets was resuspended with a similar volume of hypotonic buffer and then sonicated in an ice bath for 2 min. The two fractions in protein samples were separated by SDS-PAGE.

### 4.9. Solid-Phase Sandwich Enzyme-Linked Immunosorbent Assay (ELISA) Analysis of Secreted Aβ40 and Aβ42

The secretion of Aβ into the cell culture medium was analyzed with a Human/Rat Beta Amyloid (40) ELISA Kit and Human/Rat Beta Amyloid (42) ELISA Kit (294-64701, 290-62601, FUJIFILM Wako Pure Chemical Corporation, Osaka, Japan) as previously described [11]. The results are presented as percentages in comparison with control culture values.

### 4.10. Brain Samples

Brain hippocampal tissues from six patients with AD and five age-matched controls were obtained (Table S2). The research on human samples was reviewed and approved by the Ethics Committee of Fujian Medical University. In the vast majority of cases, samples harvested within 30 h postmortem were utilized.

### 4.11. Construction of the Protein–Protein Interaction Network

The protein–protein interaction network was constructed and analyzed using the STRING functional protein association network database (https://string-db.org) (accessed on 23 June 2022) [38].

### 4.12. Statistical Analysis

Statistical analyses were performed using GraphPad Prism software (version 8.3.0, GraphPad, San Diego, CA, USA). The data are expressed as the mean ± standard deviation (SD) of three independent experiments. The statistical significance was analyzed by either a two-tailed unpaired *t*-test or one-way ANOVA followed by Tukey’s post hoc test. Significance was set to *p* < 0.05.

## 5. Conclusions

**Figure 10:**
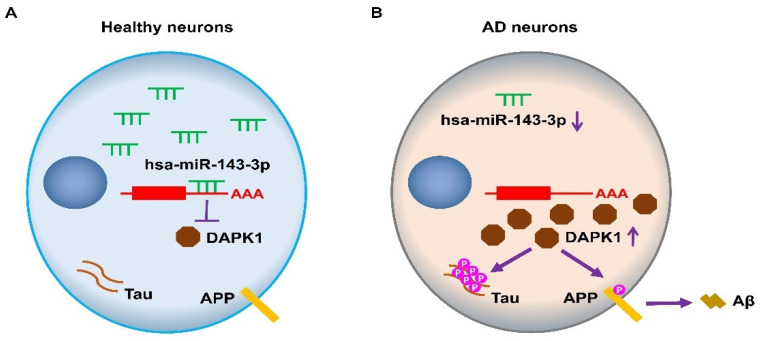
Schematic diagram showing the proposed roles of hsa-miR-143-3p in the regulation of tau phosphorylation and Aβ production through DAPK1 in AD. (**A**) In healthy neurons, hsa-miR-143-3p reduces the expression of DAPK1 by directly binding to its mRNA 3′UTR, which results in inhibition of tau phosphorylation and Aβ production. (**B**) In AD neurons, the hsa-miR-143-3p levels are downregulated, and DAPK1 expression is upregulated, resulting in increased tau phosphorylation, APP phosphorylation, and Aβ secretion.

In summary, the above-described findings demonstrate that hsa-miR-143-3p directly inhibits human DAPK1 expression, and this inhibition results in reduced tau phosphorylation, increases in neurite outgrowth and microtubule assembly, and attenuation of APP phosphorylation and Aβ production (Figure 10). Furthermore, the hsa-miR-143-3p levels are decreased and inversely correlated with DAPK1 expression in human AD brains. This study thus suggests that hsa-miR-143-3p might play critical roles in regulating both tau function and APP processing by directly targeting DAPK1 and thus offers a potential novel therapeutic strategy for AD treatment.

## Data Availability

All data generated or analyzed during this study are available from the corresponding author on reasonable request.

## Supplementary Materials

The following supporting information can be downloaded at: https://www.mdpi.com/article/10.3390/ijms23147992/s1.
