# Supplementary material for: miR-143-3p Inhibits Aberrant Tau Phosphorylation and Amyloidogenic Processing of APP by Directly Targeting DAPK1 in Alzheimer’s Disease
Source: Int J Mol Sci. 2022 Jul 20;23(14):7992. doi: 10.3390/ijms23147992 (PMC9317260; doi:10.3390/ijms23147992)
Supplement: Supplementary file 1 [file ijms-23-07992-s001.zip › ijms-1773710-supplementary.pdf]

International Journal of *Molecular Sciences*

MDPI

Article

MiR-143-3p Inhibits Aberrant Tau Phosphorylation and Amy-loidogenic Processing of APP by Directly Targeting DAPK1 in Alzheimer's Disease

Long Wang †, Xindong Shui †, Yingxue Mei, Yongfang Xia, Guihua Lan, Li Hu, Mi Zhang, Chen-Ling Gan, Ru-omeng Li, Yuan Tian, Quling Wang, Xi Gu, Dongmei Chen, Tao Zhang and Tae Ho Lee *

Fujian Key Laboratory of Translational Research in Cancer and Neurodegenerative Diseases, Institute for Translational Medicine, School of Basic Medical Sciences, Fujian Medical University, Fuzhou 350122, China

* Correspondence: tlee0813@fjmu.edu.cn or leethres@hotmail.com; Tel.: +86-591-2286-2498

† These authors contributed equally to this work.

Supplementary Materials

Table S1. Primary antibodies used in this study.

Antibodies	Dilutions	Source	Identifier
Mouse anti-β-actin	1:40000 (WB)	Sigma	Cat# A5441
Mouse anti-DAPK1	1:1000 (WB)	Sigma	Cat# D2178
Mouse anti-Tau	1:500 (WB)	Invitrogen	Cat# AHB0042
Rabbit anti-pT231-Tau	1:500 (WB)	Abcam	Cat# ab151559
Rabbit anti-pT231-Tau	1:200 (IF)	Abcam	Cat# ab151559
Rabbit anti-pS262-Tau	1:500 (WB)	Invitrogen	Cat# 44750G
Rabbit anti-pS396-Tau	1:500 (WB)	Anaspec	Cat# AS-54977
Rabbit anti-APP	1:2000 (WB)	Abcam	Cat# Ab32136
Rabbit anti-pT668-APP	1:500 (WB)	Cell Signaling Technology	Cat# 6986
Rabbit anti-Tuj1	1:200 (IF)	Beyotime	Cat# AT809
Rabbit anti-α-Tubulin	1:2000 (WB)	Cell Signaling Technology	Cat# 2125S

Abbreviations: IF, immunofluorescence; WB, Western blotting.

Table S2. Characteristics of postmortem AD patients and age-matched control human hippocampus samples.

No	NPDx	Gender	Age	PMI (h)	B&B	CERAD
1	Normal	F	83	29.40	I	A
2	Normal	F	79	17.58	I	A
3	Normal	F	66	25.00	I	A
4	Normal	F	73	20.00	I	A
5	Normal	F	77	<12.00	III	B
6	AD	F	80	22.00	VI	C
7	AD	F	80	10.00	V	C
8	AD	F	55	48.00	V-VI	C
9	AD	F	92	24.00	VI	C
10	AD	F	82	16.83	VI	C
11	AD	F	87	29.58	VI	C

Abbreviations: B&B (out of VI), Braak and Braak stage of AD; CERAD, Consortium to Establish a Registry for Alzheimer's disease; F, female; NPDx, neuropsychological diagnosis; PMI, postmortem interval.

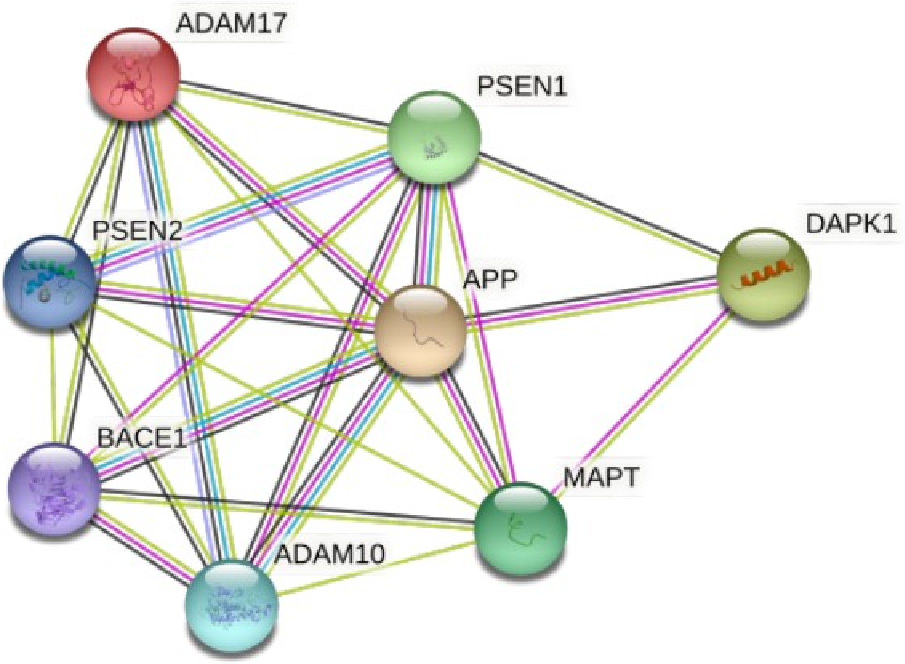

Figure S1. Protein-protein interaction network comprising DAPK1, tau (MAPT), APP, PSEN1, PSEN2, ADAM10, ADAM17, and BACE1 associated with Alzheimer's disease using the STRING database (http://string-db.org).
